# Double-walled **pyr** topology networks from a novel fluoride-bridged heptanuclear metal cluster†
†Electronic supplementary information (ESI) available: Experimental details, single-crystal XRD data, PXRD patterns, TGA curves, sorption data fit and *Q*_st_ plot. CCDC 1060661. For ESI and crystallographic data in CIF or other electronic format see DOI: 10.1039/c5sc01515d



**DOI:** 10.1039/c5sc01515d

**Published:** 2015-05-22

**Authors:** Kai-Jie Chen, John J. Perry IV, Hayley S. Scott, Qing-Yuan Yang, Michael J. Zaworotko

**Affiliations:** a Department of Chemical & Environmental Sciences , University of Limerick , Limerick , Republic of Ireland . Email: Michael.Zaworotko@ul.ie

## Abstract

Two isostructural metal–organic materials, **Tripp-1-M** (**Tripp** = 2,4,6-tris(4-pyridyl)pyridine; M = Co, Ni), that exhibit binodal 3,6-connected **pyr** network topology have been prepared and characterized. **Tripp-1-M** are based upon a novel M_7_F_12_^2+^ cluster that possesses 12 connection points but, because of double cross-linking by 3-connected **Tripp** ligands, it functions as a 6-connected supermolecular building block (SBB).

## Abstract 2

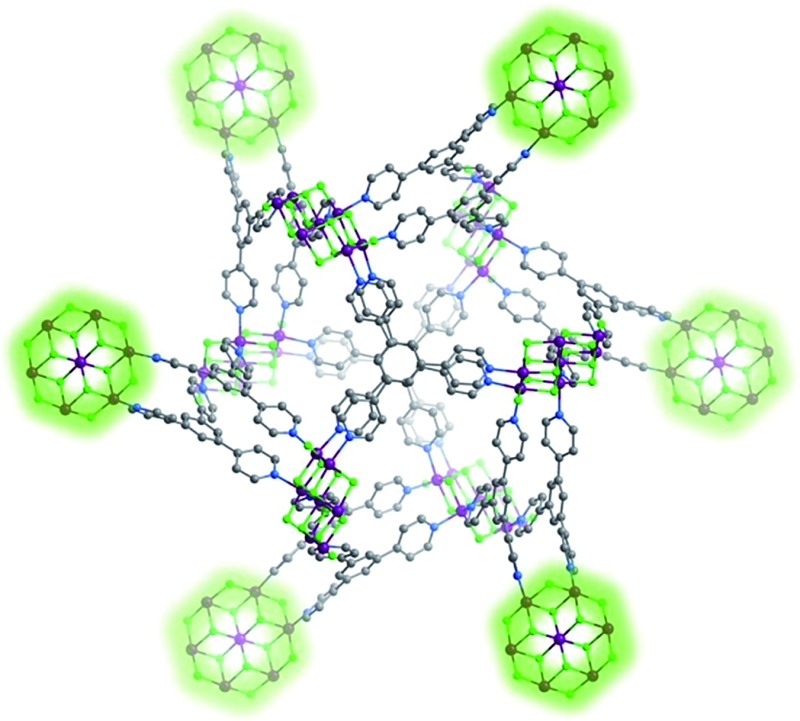
Two isostructural networks with double-walled **pyr** topology comprised of novel fluoride-bridged heptanuclear metal clusters and 3-connected ligands have been synthesized and characterized by X-ray diffraction, thermogravimetric analysis, and gas sorption experiments.

## Introduction

Metal–organic materials (MOMs)^1^ have attracted rapidly increasing attention from the scientific community in the last two decades thanks to their inherent modularity. This modularity can promote diversity of composition, amenability to systematic design^2^ and control over certain bulk properties.^3^ However, not all MOMs are well suited to serve as prototypal platforms for the generation of families of materials with the same topology. Such platforms are important because they enable systematic fine-tuning of both pore size (*e.g.* organic linkers with different lengths) and pore chemistry (*e.g.* functional group substitution at the linker or metal substitution at the node). Most platforms are built from single metal ion or small cluster (molecular building block, MBB) nodes and are exemplified by platforms sustained by carboxylate clusters such as 4-connected (4-c) M_2_(RCOO)_4_ (*e.g.* **HKUST-1**,^4^ PCN-6,^5^ MOF-2,^6^ DMOF-1 ^7^ and NU-100 ^8^), 6-c M_3_O(RCOO)_6_ (*e.g.* MIL-88,^9^ MIL-101 ^10^ and PCN-600 ^11^) and 6-c M_4_O(RCOO)_6_ (*e.g.* **MOF-5** ^12^ and MOF-177 ^13^). The exploitation of larger, high symmetry clusters offers the possibility of much higher levels of connectivity and even greater control over topology. Such “supermolecular building blocks”, SBBs, are exemplified by 12-connected (12-c) Zr_6_O_4_(OH)_4_(RCOO)_12_ ^14^ and 24-c “nanoball” Cu_24_(1,3-bdc)_24_ clusters.^15^ High connectivity mixed carboxylate/N-donor clusters have also been utilised in this context.^16^ In addition to affording greater control over topology because of fewer possible topological outcomes, higher connectivity nodes can result in greater robustness.^17^

**Fig. 1:**
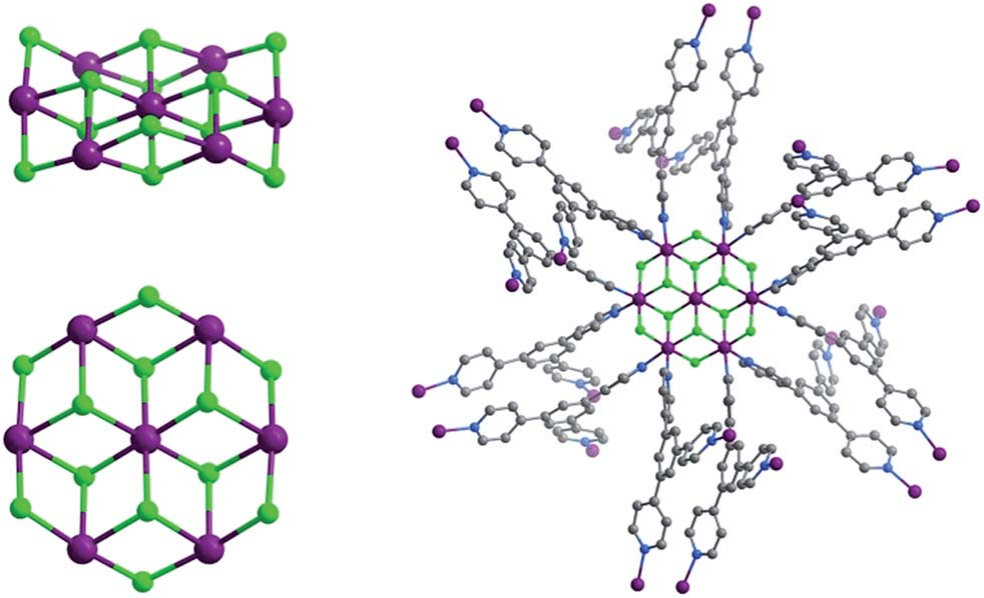
(Left) Perspective and above views of the novel M_7_F_12_^2+^ cluster that sustains **Tripp-1-M**. (right) Illustration of the 12 connection points of the Co_7_F_12_ SBB in **Tripp-1-Co** (Co, F, N and C atoms in purple, green, blue and grey). Solvent molecules, hydrogen atoms and counter-ions are omitted for the sake of clarity.

**Scheme 1:**
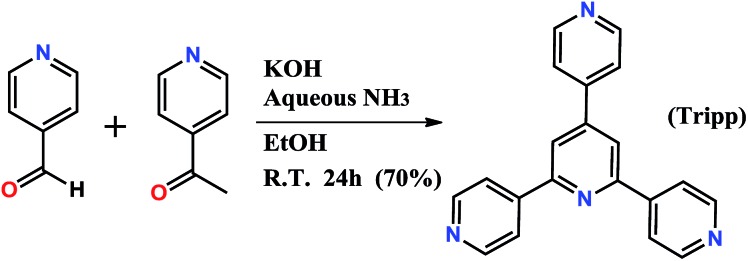
Synthesis of 2,4,6-tris(4-pyridyl)pyridine (**Tripp**).

**Fig. 2:**
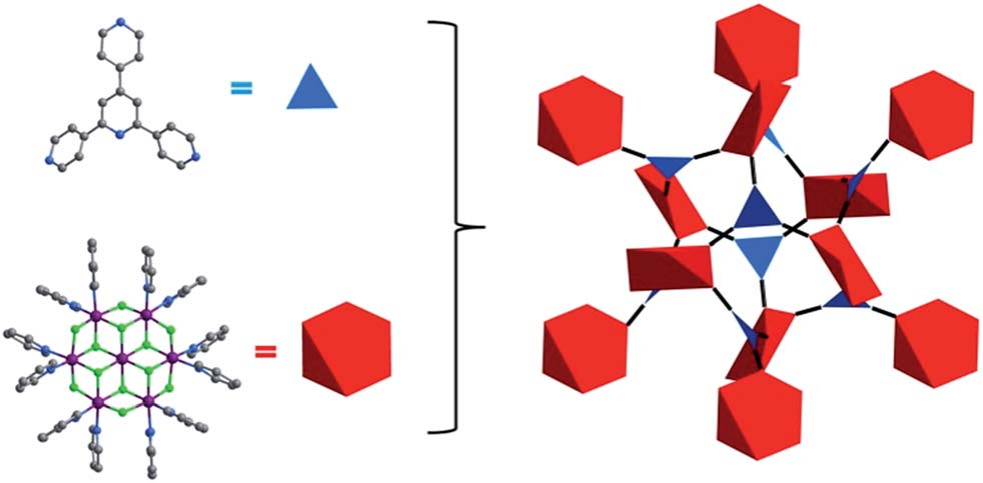
Illustration of the topology of **Tripp-1-M**. The **Tripp** ligand is represented by a 3-c triangle (blue) whereas the M_7_F_12_^2+^ SBB is reduced to a 6-c octahedron (red).

In this contribution, we introduce a new inorganic SBB of formula M_7_F_12_^2+^ (M = Co, Ni, Fig. 1) and demonstrate that it can serve as a 6-c SBB through double cross-linking of its 12 connection points by a facile to prepare 3-c ligand, 2,4,6-tris(4-pyridyl)pyridine, **Tripp** (Scheme 1). A new type of double-walled **pyr** topology (Fig. 2) material which exhibits permanent porosity is thereby generated.

In previous work, double^18,19^ or quadruple^20^ cross-linking of carboxylate^21^ or oxide^22^ based SBBs has been shown to represent a suitable approach to build MOMs with well-known^23^ or hitherto novel^24^ topology. However, the use of fluoride-bridged MBBs and SBBs as nodes for the construction of three-dimensional MOMs remains rare.^25^ This is despite the fact that discrete fluoride-bridged metal clusters are known^26,27^ and that such structures can exhibit interesting magnetic properties.^28^

## Results and discussion

**Fig. 3:**
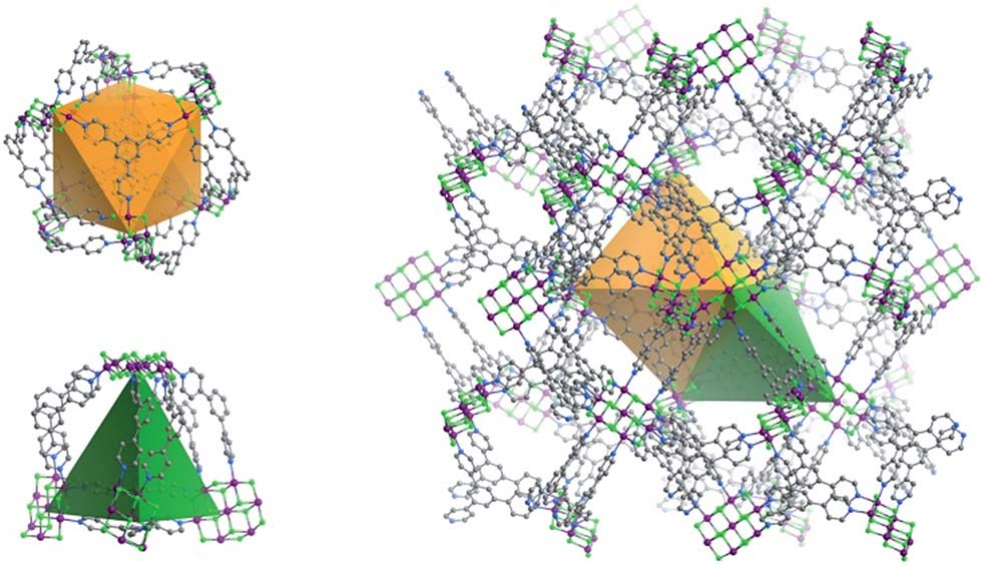
The two types of double-walled cages (OC, left above; TC left, below) found in **Tripp-1-M** (right).

**Tripp** was prepared by the cyclization reaction of 4-acetylpyridine and 4-pyridinecarbaldehyde (Scheme 1).^29^ Single crystals of **Tripp-1-Co** were initially obtained by solvothermal reaction between Co(NO_3_)_2_·6H_2_O, **Tripp** and (NH_4_)_2_SiF_6_ in DMF (for full details of synthetic procedures see ESI†). **Tripp-1-Co** crystallizes in the cubic space group *Pa*3. A crystallographic 3-fold axis runs through the centre of the **Tripp** node and the disordered atoms of the central pyridine ring were therefore refined as 2/3 carbon and 1/3 nitrogen. All atoms of central pyridine ring are presented as carbon atoms for clarity in Fig. 1 and 3. In **Tripp-1-Co**, every **Tripp** ligand links three M_7_F_12_^2+^ SBBs and every SBB is connected by 12 **Tripp** ligands. However, the arrangement of the 12 connection points enables double cross-linking by pairs of **Tripp** ligands (Fig. 1) meaning that the connectivity is effectively reduced to 6. Therefore, each pair of **Tripp** ligands can be simplified to a single node, the M_7_F_12_ SBB can be treated as a 6-c node and the **Tripp** ligand as a 3-connected node. The outcome of this connectivity is structure that exhibits binodal 3,6-connected **pyr** topology (Fig. 2), of which there are relatively few examples^22b,30^ when compared to other types of 3,6 nets such as those with **rtl**, **ant**, **sit** or **qom** topology.

The structure of **Tripp-1-M** is comprised of two types of double-walled cages; tetrahedral cages (TCs) and octahedral cages (OCs) with face-sharing configurations (Fig. 3). The M_7_F_12_^2+^ SBB is located at the vertex of every TC and OC, while the double-walls are constructed from **Tripp** ligands. The pore diameter of the TC and OC are *ca.* 7.7 and 7.6 Å, respectively. Every OC is surrounded by eight adjacent TCs and every TC is surrounded by four OCs. However, there are two faces and one face capped by double walls of **Tripp** ligands in the OCs and TCs, respectively. Therefore, each OC connects with only six TCs and each TC crosslinks with three OCs, which corresponds to the required 3,6-connectivity of a **pyr** net. The guest-accessible porosity (considering the presence of counter ions) of **Tripp-1-Co** is 53.5%, based on Platon software.^31^ Two other structures with **pyr** topology using similar tripyridyl-based 3-c ligands, 2,4,6-tri(4-pyridyl)-1,3,5-triazine (TPT) and 1,3,5-benzene tricarboxylic acid tris[*N*-(4-pyridyl)amide (TPBTC), with 6-c metal ions Hg^2+^ and Cd^2+^, were reported by Robson and Kitagawa, respectively.^32^ These tripyridyl-based ligands can also be utilized as 3-c organic nodes to construct nets with high porosity.^33^ Further, some discrete cages consisting of 4-c Pd^2+^ ions linked by TPT and cage-based three-dimensional nets were reported by Fujita and co-workers.^34^ In contrast, the facile to prepare **Tripp** ligand has not been as widely studied as TPT and TPBTC.

The double-walled nature of **Tripp-1-M** is unusual and, to our knowledge, such as structure has not been prepared using a single ligand and a single SBB. However, Bu *et al.* recently reported two isostructural double-walled cage-based MOMs that were designed using a strategy based upon size-matching between two tritopic ligands (TPT and 2,4,6-tris[1-(3-carboxylphenoxy)-yl-methyl]mesitylene) connected by with same paddle-wheel unit.^18^

**Fig. 4:**
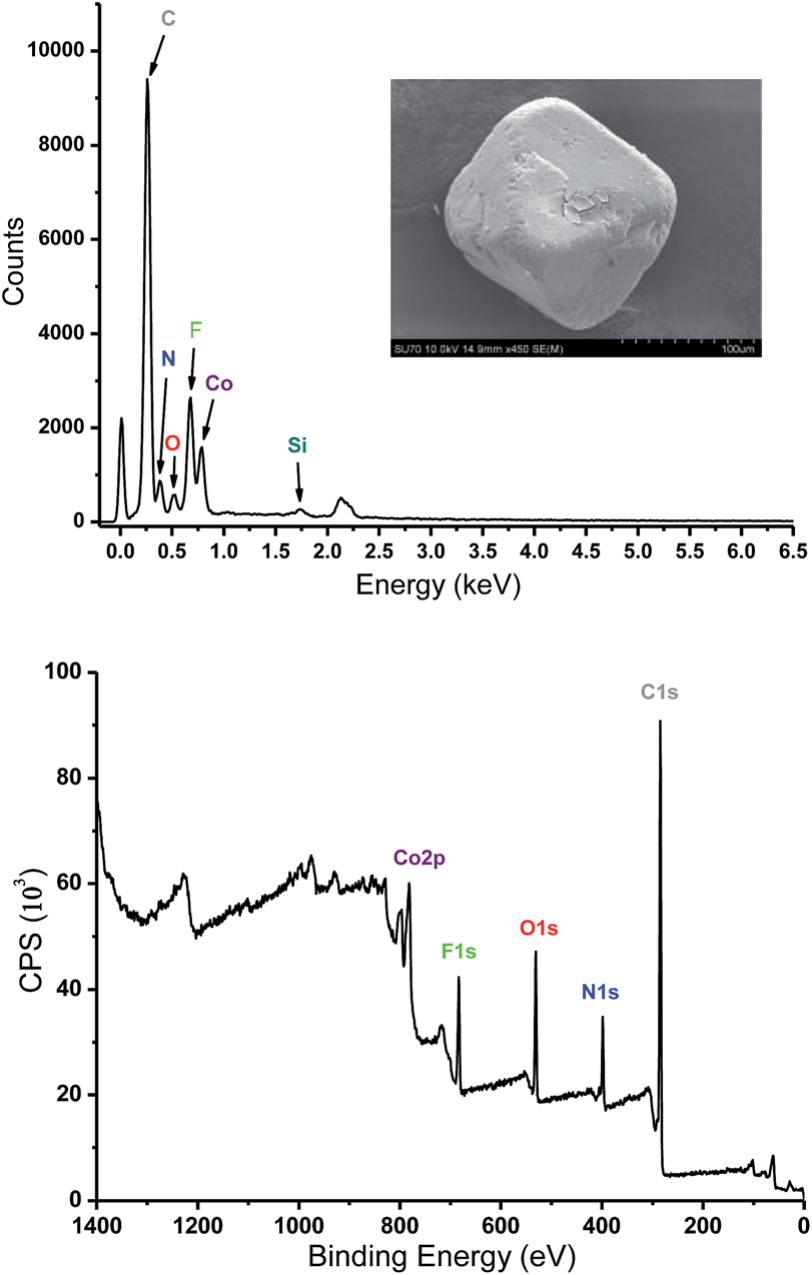
Energy-dispersive X-ray spectroscopy (top) and X-ray photoelectron spectroscopy (bottom) of **Tripp-1-Co**.

Analysis of crystal structure of **Tripp-1-Co** revealed that the Co–F distances in heptanuclear cluster lie the range from 2.036 (5) to 2.121 (7) Å, which are consistent with the values found in other fluoride-bridged Co(ii) structures, *e.g.* [Co_5_F_2_(tetrazolate)_4_(H_2_O)_4_]^35^ and [Co_12_(RCOO)_6_(PO_4_)_4_F_4_(H_2_O)_6_](NO_3_)_2_.^36^ The crystal structure of **Tripp-1-Co** also revealed that the charge of each heptanuclear cobalt cluster is balanced by one SiF_6_^2–^ anion that exhibits three-fold F···F (distance: 2.68 (2) Å) interactions with three bridging F^–^ anions (Fig. S1†). Energy-dispersive X-ray spectroscopy verified the presence of Si and F in crystals of **Tripp-1-Co** (Fig. 4). There are previous reports concerning the generation of F^–^ by decomposition of PF_6_^–^ and BF_4_^–^,^26,37^ so we speculated that the source of the bridging fluoride anions in the Co_7_F_12_^2+^ SBBs was *in situ* decomposition of SiF_6_^2–^. Further, there are two drawbacks to the use of this synthetic method: the relatively low solubility of (NH_4_)_2_SiF_6_ in DMF; the requirement to decompose SiF_6_^2–^ anions before the SBB can form. These drawbacks mean that unreacted (NH_4_)_2_SiF_6_ is isolated in a physical mixture with **Tripp-1-Co** crystals, mitigating against phase purity and also resulting in low product yield (*ca.* 10%). Therefore, we tested a different synthetic approach involving reflux of starting materials in DMF/MeOH with NH_4_F instead of (NH_4_)_2_SiF_6_ as the F^–^ source. This method facilitated an increase in yield to 80%. The composition of **Tripp-1-Co** was changed since SiF_6_^2–^ counterions are no longer present. Rather, two NO_3_^–^ anions from the Co(NO_3_)_2_ starting material balance charge as indicated by the presence of two diagnostic peaks measured using FT-IR at around 1320 and 1400 cm^–1^ (Fig. S4†).^38^ The relatively high solubility of NH_4_F in MeOH enabled subsequent isolation of pure reaction product. The purity of bulk product was established by powder X-ray diffraction (PXRD) patterns of as-synthesized samples, which are good matches to those calculated from the crystal structure of **Tripp-1-Co** (Fig. S5†). To further verify the composition of the M_7_F_12_^2+^ cluster, X-ray photoelectron spectroscopy (XPS) analysis of a sample prepared from NH_4_F was conducted, and a molar ratio for F : Co of 1.44 (expected 1.71) was observed (Fig. 4). A series of control experiments conducted without using NH_4_F as a source of F^–^ revealed that different concentrations of metal and ligand and different reaction temperatures failed to afford the desired **Tripp-1-Co** product. Nevertheless, these experiments confirm the essential role that F^–^ plays in construction of the M_7_F_12_^2+^ SBB and subsequently the overall MOM framework. The isostructural nickel analogue of **Tripp-1-Co**, **Tripp-1-Ni**, was obtained *via* the same modified synthetic protocol with a yield of *ca.* 70% as verified by PXRD (Fig. S6†).

To address the thermal stability of **Tripp-1-M**, thermo-gravimetric analysis (TGA) was conducted for both as-synthesized and MeOH exchanged samples. The resulting TGA plots reveal that solvent guest molecules in the as-synthesized samples can be fully exchanged with MeOH, which in turn can be removed below 110 °C. No further weight loss until after 300 °C was observed, which we presume is a consequence of framework decomposition (Fig. S8†). Framework integrity was also verified by PXRD experiments conducted after desolvation of MeOH-exchanged samples at 120 °C. Furthermore, samples exposed to the air under ambient conditions for two months, were observed to exhibit PXRD patterns conforming to those calculated from single-crystal data (Fig. S5 and S6†). These results demonstrate that both **Tripp-1-Co** and **Tripp-1-Ni** possess good thermal and air/moisture stability, which we attribute to some extent to the high connectivity of the M_7_F_12_^2+^ SBB.

**Fig. 5:**
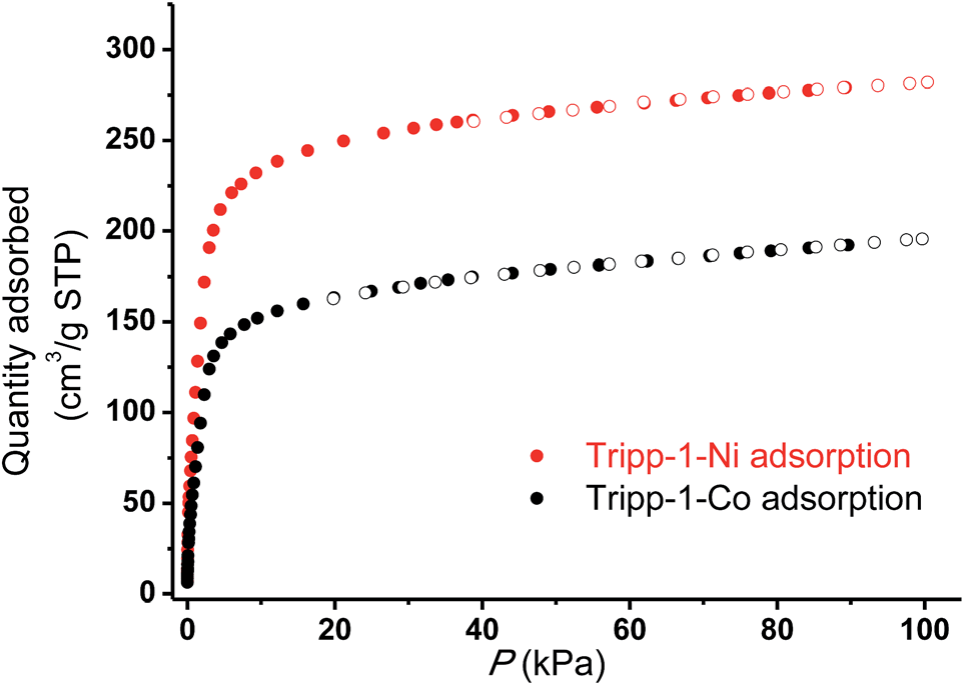
CO_2_ adsorption isotherms (filled symbols) and desorption (empty symbols) for **Tripp-1-Co** (black) and **Tripp-1-Ni** (red) conducted at 195 K.

**Fig. 6:**
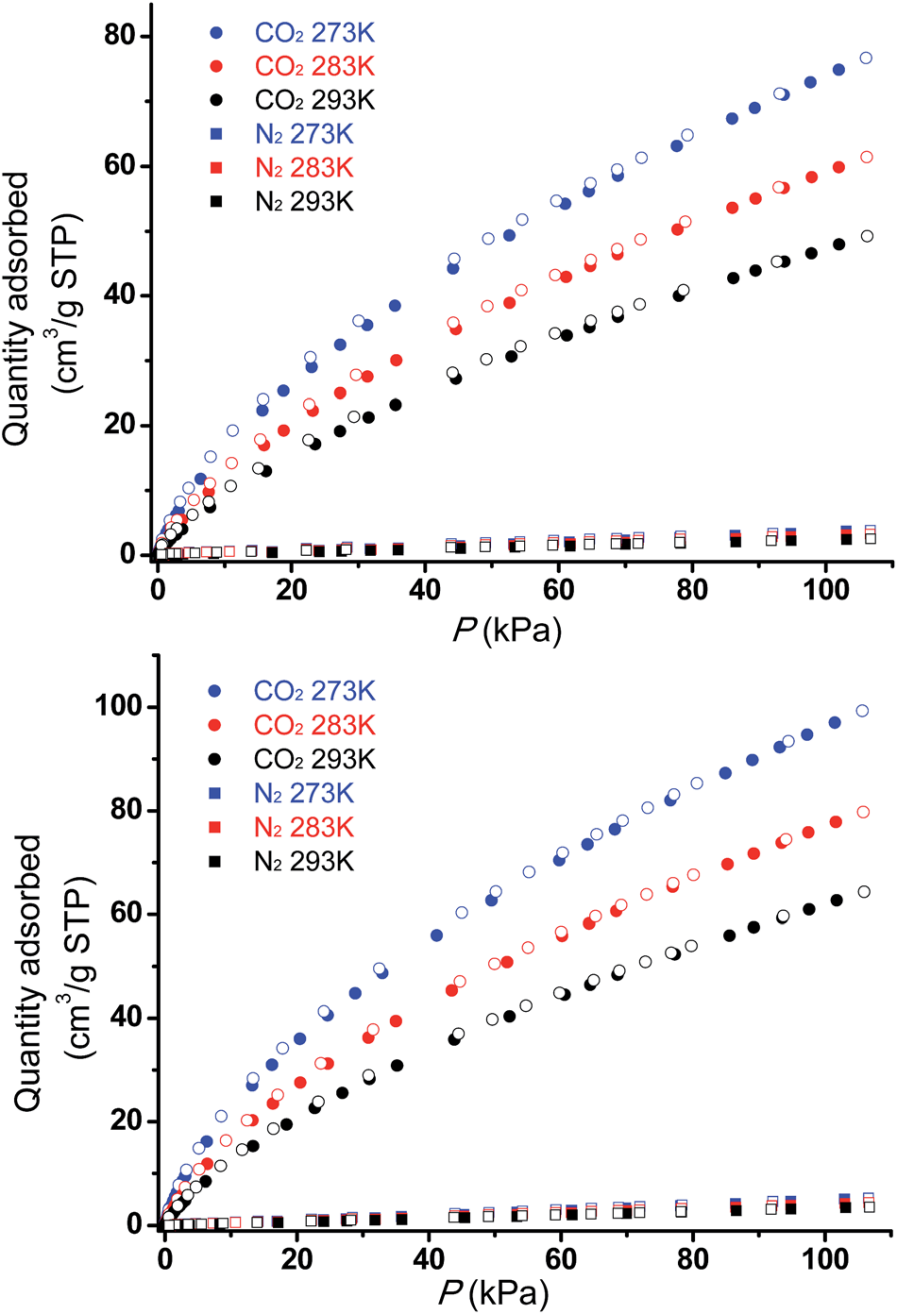
CO_2_ and N_2_ adsorption (filled symbols) and desorption (empty symbols) isotherms for **Tripp-1-Co** (top) and **Tripp-1-Ni** (bottom) at three temperatures, 273 K, 283 K and 293 K.

The permanent porosities of **Tripp-1-Co** and **Tripp-1-Ni** were established by measuring CO_2_ sorption isotherms at 195 K (Fig. 5). The apparent BET surface area was calculated to be 822 and 1149 m^2^ g^–1^ for **Tripp-1-Co** and **Tripp-1-Ni**, respectively. Pore volumes of 0.358 and 0.516 cm^3^ g^–1^ for **Tripp-1-Co** and **Tripp-1-Ni** were calculated by assuming liquid filling of CO_2_ at saturated state, which are close to the value of 0.587 cm^3^ g^–1^ estimated from the crystal data for **Tripp-1-Co**. The relatively lower uptake of **Tripp-1-Co** might be attributed to partial collapse of the framework during activation, which does not appear to occur for **Tripp-1-Ni**. CO_2_ and N_2_ sorption isotherms of **Tripp-1-M** at 273, 283 and 293 K were also measured. As shown in Fig. 6, CO_2_ uptakes of 76.7, 61.4 and 49.2 cm^3^ g^–1^ at 273, 283 and 293 K, respectively, are much higher than the values of 3.7, 3.2 and 2.5 cm^3^ g^–1^ observed for N_2_ in **Tripp-1-Co**. **Tripp-1-Ni** exhibits higher CO_2_ uptakes of 99.3, 79.8 and 64.3 cm^3^ g^–1^ at 273, 283 and 293 K, respectively, than those observed for **Tripp-1-Co**. Meanwhile, N_2_ uptakes at 273, 283 and 293 K are only 5.2, 4.3 and 3.5 cm^3^ g^–1^ for **Tripp-1-Ni**. The high CO_2_ uptakes of **Tripp-1-M** could be attributed to high pore volume and polarized pore surface originating from the heptanuclear cluster and central pyridine ring of the **Tripp** ligand. These results indicate that both **Tripp-1-M** variants exhibit good selectivity for CO_2_ over N_2_. Preliminary CO_2_/N_2_ selectivity for **Tripp-1-Co** and **Tripp-1-Ni**, calculated from the uptakes of CO_2_ at 0.15 bar and N_2_ at 0.85 bar, are 41.4 and 40.8, 36.8 and 36.7, and 36.2 and 32.9 at 273, 283 and 293 K. These uptakes and selectivities are comparable to many well-known MOMs containing polar functional groups and/or open metal sites, both of which are absent in **Tripp-1-M** materials.^39^

To assess the strength of interaction between CO_2_ and framework, the CO_2_ isotherms measured at 273, 283 and 293 K were fitted using the virial equation (Fig. S9†), and the isosteric heat of adsorption (*Q*_st_) was calculated using the Clausius–Clapeyron equation. The enthalpies at zero loading for **Tripp-1-Co** and **Tripp-1-Ni** are 25.6 and 26.3 kJ mol^–1^, respectively (Fig. S10†). These values are also consistent with those observed in other classes of MOMs such as **MOF-5** (34 kJ mol^–1^),^40^ **HKUST-1** (35 kJ mol^–1^),^41^ **MAF-25** (26 kJ mol^–1^),^42^ **InOF-1** (29 kJ mol^–1^),^43^ **NOTT-140** (25 kJ mol^–1^).^44^

## Conclusions

In summary, we report a novel fluoride-bridged heptanuclear metal cluster-based SBB which has not been previously observed as a discrete structure. This cluster has 12-connection points, but 3-c **Tripp** ligands doubly cross-link to adjacent SBBs in order to form **Tripp-1-M**, two isostructural MOMs with binodal 3,6-connected **pyr** network topology. Good thermal and air/moisture stabilities were observed and gas sorption experiments demonstrate that both **Tripp-1-Co** and **Tripp-1-Ni** exhibit permanent porosity. The novel M_7_F_12_^2+^ SBB reported herein has the potential to serve as an SBB for a wider range of MOMs with tailored pore sizes and surface chemistries. Follow-on studies on this platform will address properties related to gas sorption, catalysis and magnetism and are currently underway.
