# Supplementary material for: Double-walled pyr topology networks from a novel fluoride-bridged heptanuclear metal cluster
Source: Chem Sci. 2015 May 22;6(8):4784–9. doi: 10.1039/c5sc01515d (PMC5502393; doi:10.1039/c5sc01515d)
Supplement: Supplementary file 1 [file SC-006-C5SC01515D-s001.pdf]

Electronic Supplementary Material (ESI) for Chemical Science.
This journal is © The Royal Society of Chemistry 2015

Double-Walled pyr Topology Networks from a Novel Fluoride-Bridged Heptanuclear Metal Cluster

*Kai Jie Chen, John J. Perry IV, Hayley S. Scott, Qing-Yuan Yang and Michael J. Zaworotko**

Department of Chemical & Environmental Sciences, University of Limerick, Limerick, Republic of Ireland

E-mail: Michael.Zaworotko@ul.ie

Supplementary Index

<u>**Experimental Section**</u>

<u>**X-Ray crystallography**</u>

Table 1. Crystal Data and Structure Refinement for Tripp-1-Co

Figure S1. Short F…F interaction in Tripp-1-Co

Figure S2. The packing views along *a* and [111] axis

Figure S3. X-ray photoelectron spectroscopy of Tripp-1-Co sample from the reflux reaction

Figure S4. Infrared Spectrogram of Tripp-1-Co and Tripp-1-Ni samples from the reflux reactions

Figure S5. The powder X-ray diffraction patterns at different conditions of Tripp-1-Co

Figure S6. The powder X-ray diffraction patterns at different conditions of Tripp-1-Ni

Figure S7. The powder X-ray diffraction patterns after sorption measurements

Figure S8. Thermo gravimetric analysis of Tripp-1-Co and Tripp-1-Ni

Figure S9. The Virial fit of CO_2 isotherms of Tripp-1-Co and Tripp-1-Ni

Figure S10. The isosteric heats of adsorption of Tripp-1-Co and Tripp-1-Ni

Experimental Section

Materials and Physical Measurements. Commercially available reagents were used as received without further purification. Thermo-gravimetric (TG) analyses were performed under N_2 using a TA Q50 system. Powder X-ray diffraction (PXRD) patterns were recorded on a PANalytical X'Pert MPD Pro using Cu Kα radiation with a 1D X'Celerator strip detector. Gas sorption isotherms were measured using Micromeritics Tristar II 3030 and 3Flex 3500 surface characterization analyzers. The two MeOH-exchanged samples were desolvated under high vacuum at room temperature for 16 h to remove the MeOH molecules in the pores before the measurements.

Synthesis of single crystal sample [Co_7F_{12}(Tripp)$_4$](SiF_6)·g: A mixture of $Co(NO_3)_2 \cdot 6H_2O$ (0.07 mmol, 0.021 g), **Tripp** (0.04 mmol, 0.012 g), $(NH_4)_2SiF_6$ (0.07 mmol, 0.012 g) and DMF (3 mL) was added to a 10-mL glass vial, and then the vial capped tightly was put into the oven at 120 ^oC for 48 h, which was then cooled to room temperature. After rinsed several times with fresh DMF, orange single octahedral-shaped crystals were gained for single crystal X-ray diffraction experiment.

Synthesis of [Co_7F_{12}(Tripp)$_4$](NO_3)$_2$·g (Tripp-1-Co): A mixture of $Co(NO_3)_2 \cdot 6H_2O$ (2.8 mmol, 0.814 g), Tripp (1.6 mmol, 0.496 g), NH_4F (4.8 mmol, 0.177 g), DMF (120 mL) and MeOH (30 mL) was added to a 250-mL round-bottom flask, and then reacted in reflux at 120 ^oC for 48 h, which was then cooled to room temperature. After decanting the mother liquor, the orange crystalline product dried in the air to give the yield of ca. 80% (based on Co). For gas sorption measurements, the as-synthesized sample was rinsed two times daily with fresh MeOH (100 mL × 3) for 4 days to get the MeOH-exchanged **Tripp-1-Co**. Elemental Analysis (%) for $C_{113}H_{188}N_{18}O_{57}F_{12}Co_7$ calcd: C, 40.1; H, 5.6; N, 7.5. Found: C, 38.6; H, 5.8; N, 7.8. IR: ν_{max} = 3370, 3267, 2830, 1650, 1603, 1560, 1538, 1505, 1404, 1337, 1220, 1066, 1041, 1023, 828 cm^{-1}.

[Ni_7F_{12}(Tripp)$_4$](NO_3)$_2$·g (Tripp-1-Ni): This compound was prepared in a similar procedure as **Tripp-1-Co**, by using $Ni(NO_3)_2$ (2.8 mmol, 0.816 g) in place of $Co(NO_3)_2 \cdot 6H_2O$. The resulting green crystalline product of **Tripp-1-Ni** was obtained in yield of 70%. The same exchanging procedure as

Tripp-1-Co was taken for the MeOH-exchanged **Tripp-1-Ni**. Elemental Analysis (%) for $C_{108}H_{168}N_{18}O_{52}F_{12}Ni_7$ calcd: C, 40.2; H, 5.2; N, 7.8. Found: C, 39.1; H, 5.3; N, 8.1. IR: ν_{max} = 3385, 3081, 2820, 1604, 1539, 1506, 1470, 1400, 1318, 1220, 1066, 1042, 1024, 828, 669 cm^{-1}.

X-Ray crystallography

Diffraction intensities were collected on a Bruker Quest diffractometer equipped with a CMOS detector and 1μS microfocus X-ray source Cu Kα λ = 1.5418 Å). Absorption corrections were applied by using the multi-scan program SADABS. The structures were solved with the direct method and refined with the full-matrix least-squares technique with the SHELXTL program package[1]. All nonhydrogen atoms were refined with anisotropic displacement parameters. Hydrogen atoms were generated geometrically. Crystallographic data were summarized in Table 1.

Table 1. Crystal Data and Structure Refinement for **Tripp-1-Co**

Complex	**Tripp-1-Co**		
Formula	$C_{80}H_{56}Co_7F_{18}N_{16}O_{18}Si$	Z	4
Formula weight	2312.01	D_c /g cm^{-3}	1.043
Temperature (K)	100(2)	reflns coll.	136375
Crystal system	cubic	unique reflns	2575
Space group	*Pa*-3	R_{int}	0.1192
a/Å	24.5063(4)	R_1 [$I > 2\sigma(I)$][a]	0.0990
b/Å	24.5063(4)	wR_2 [$I > 2\sigma(I)$][b]	0.2948
c/Å	24.5063(4)	R_1 (all data)	0.1276
β/°	90	wR_2 (all data)	0.3245
V/Å^3	14717.5(4)	GOF	1.026

[a] $R_1 = \Sigma||F_o|-|F_c||/\Sigma|F_o|$, [b] $wR_2 = [\Sigma w(F_o^2-F_c^2)^2/\Sigma w(F_o^2)^2]^{1/2}$

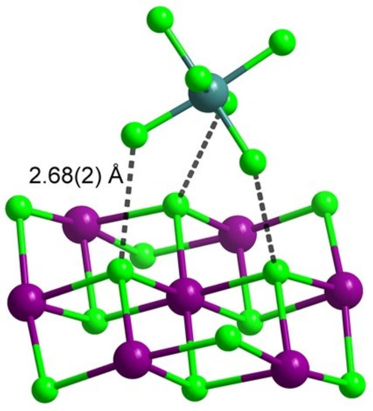

Figure S1. Short F…F interaction in Tripp-1-Co

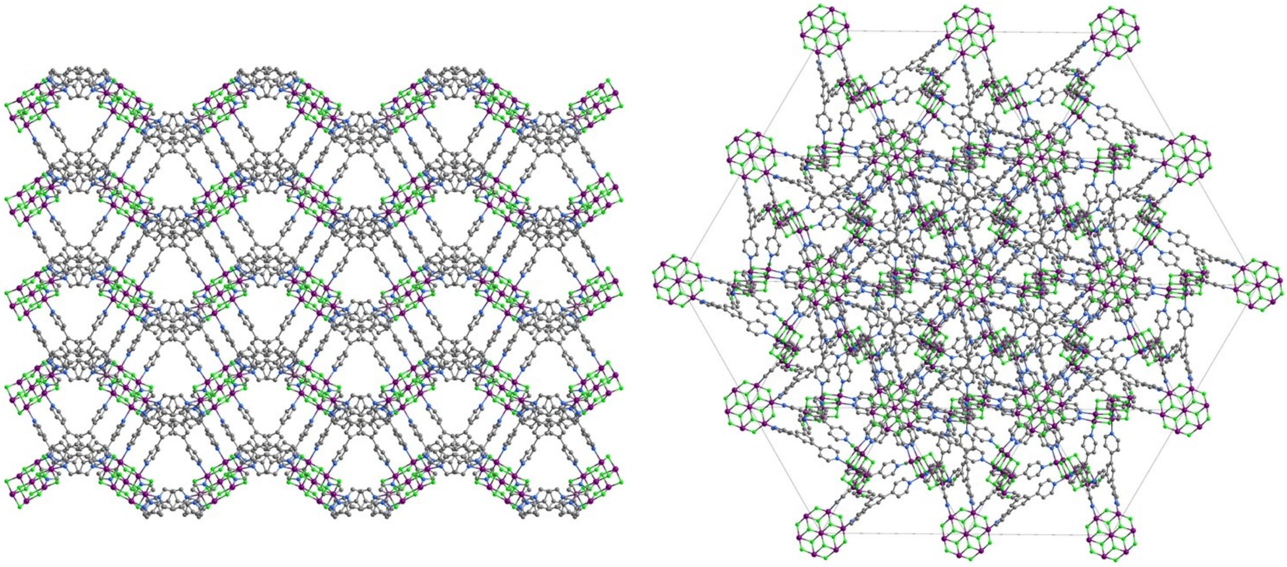

Figure S2. The packing views along *a* and [111] axis

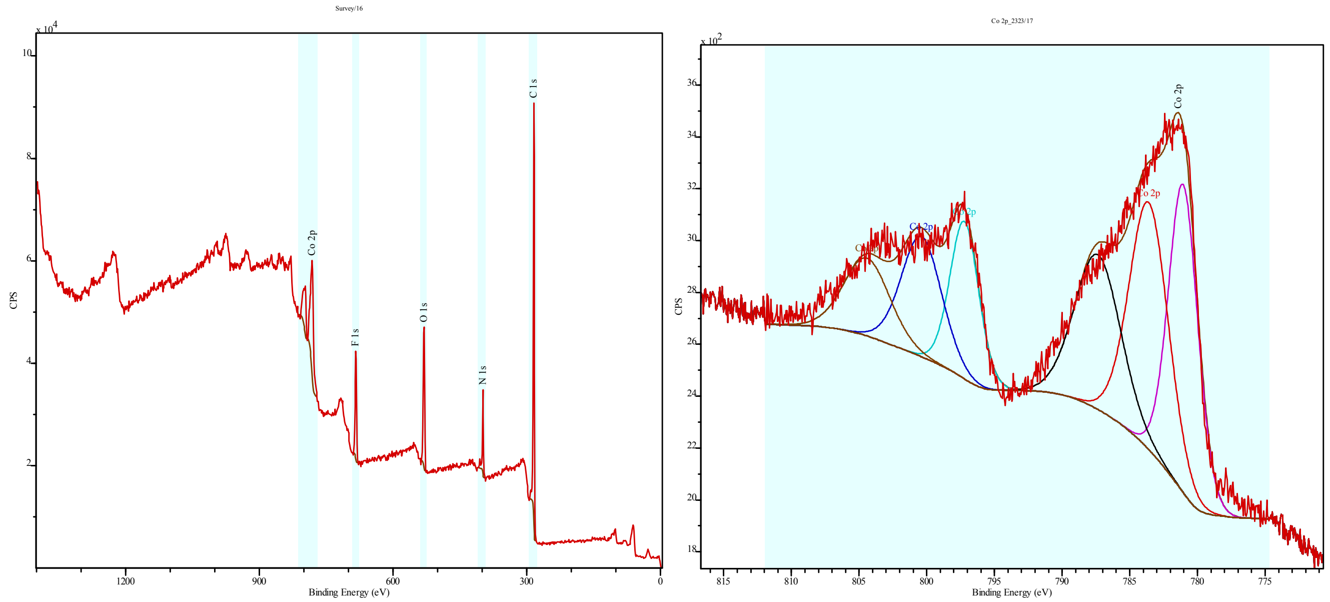

Quantification from survey spectrum		
Element	Position (eV)	Molor ratio %
O1s	530.7	9.9
C1s	284.7	71.8
N1s	398.7	8.4
F1s	683.7	5.9
Co2p	781.7	4.1

Figure S3. X-ray photoelectron spectroscopy of Tripp-1-Co sample from the reflux reaction, showing the different ratios of O, C, N, F, Co. The position of 781.7 eV for Co verifies the valence state of +2.

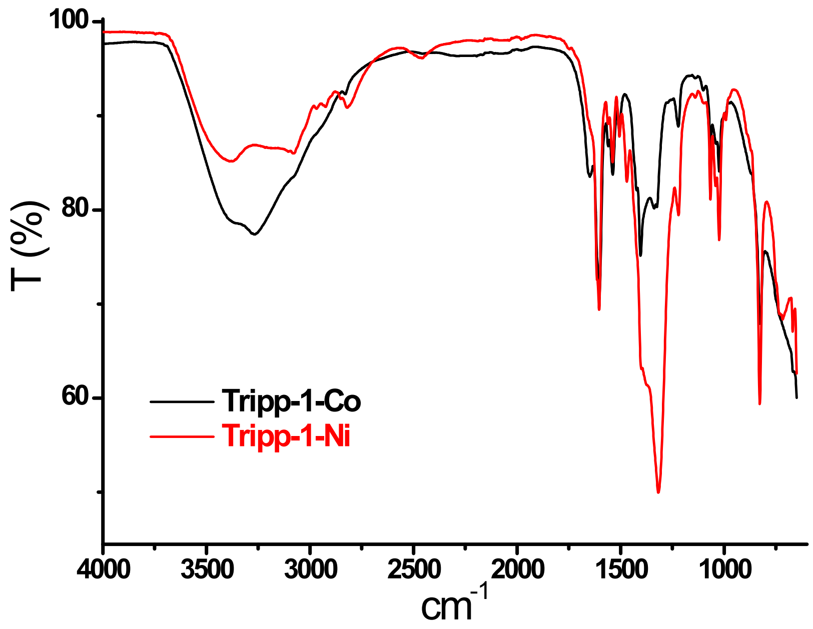

Figure S4. Infrared Spectrogram of Tripp-1-Co and Tripp-1-Ni samples from the reflux reactions.

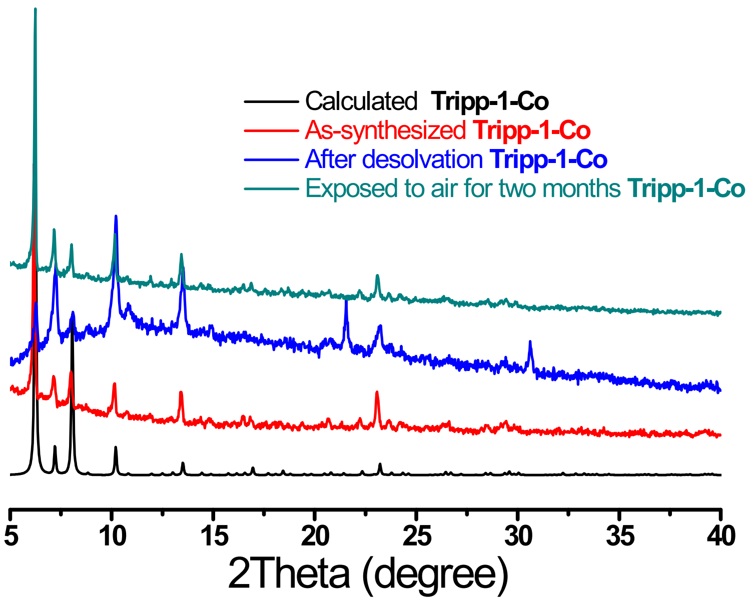

Figure S5. The powder X-ray diffraction patterns at different conditions of Tripp-1-Co

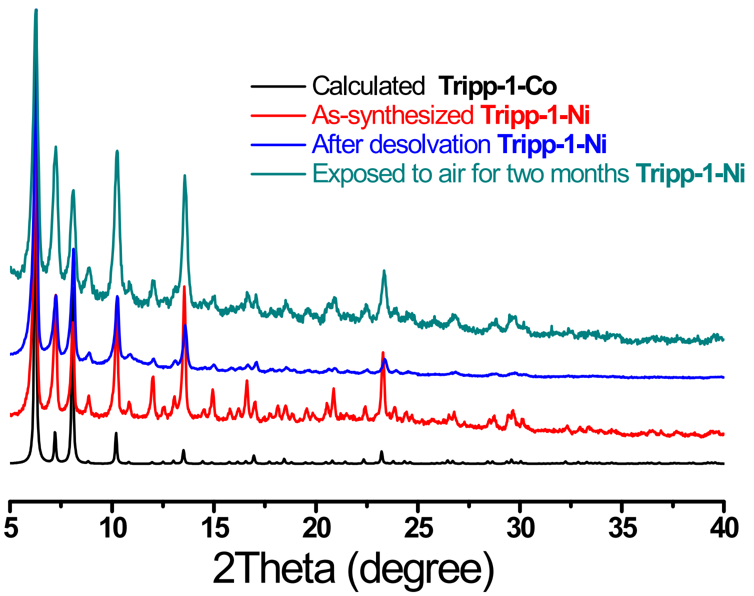

Figure S6. The powder X-ray diffraction patterns at different conditions of Tripp-1-Ni

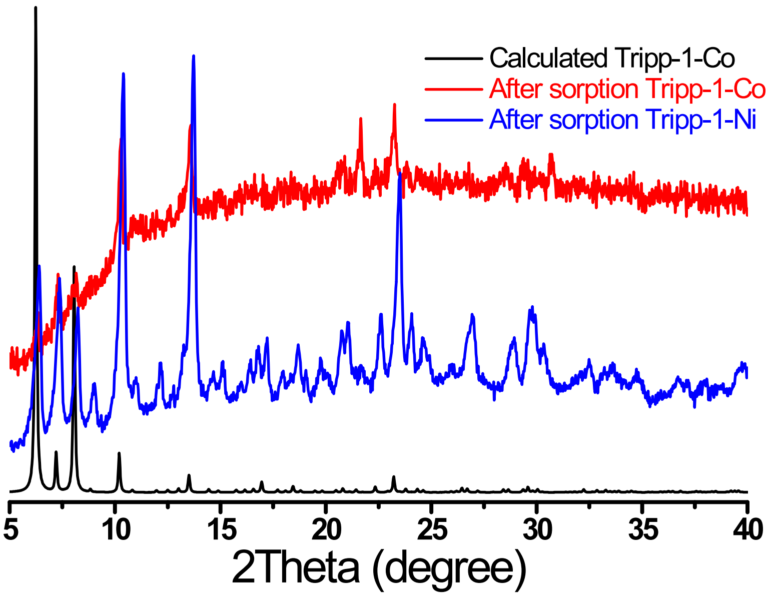

Figure S7. The powder X-ray diffraction patterns after sorption measurements

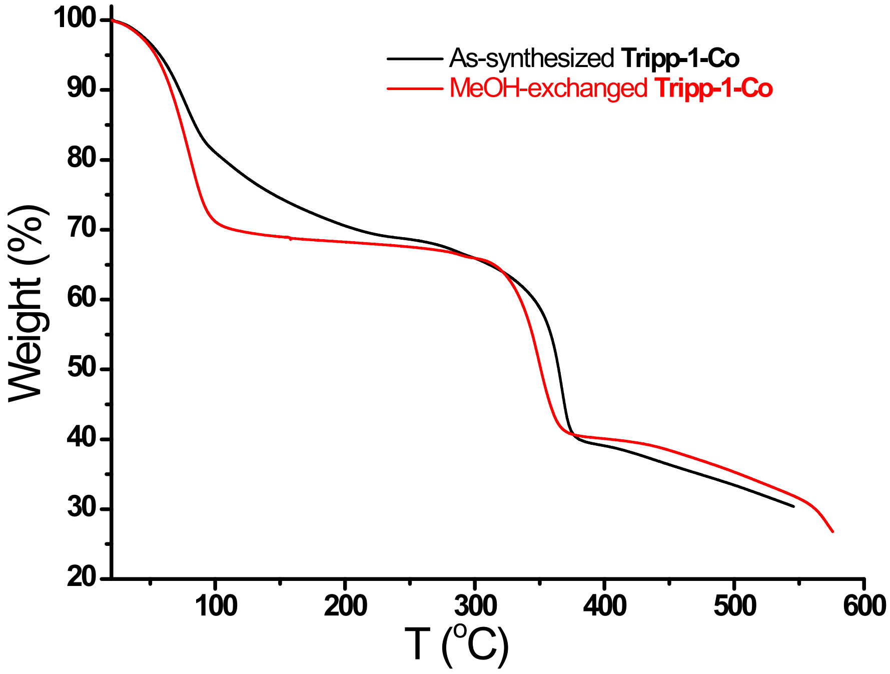

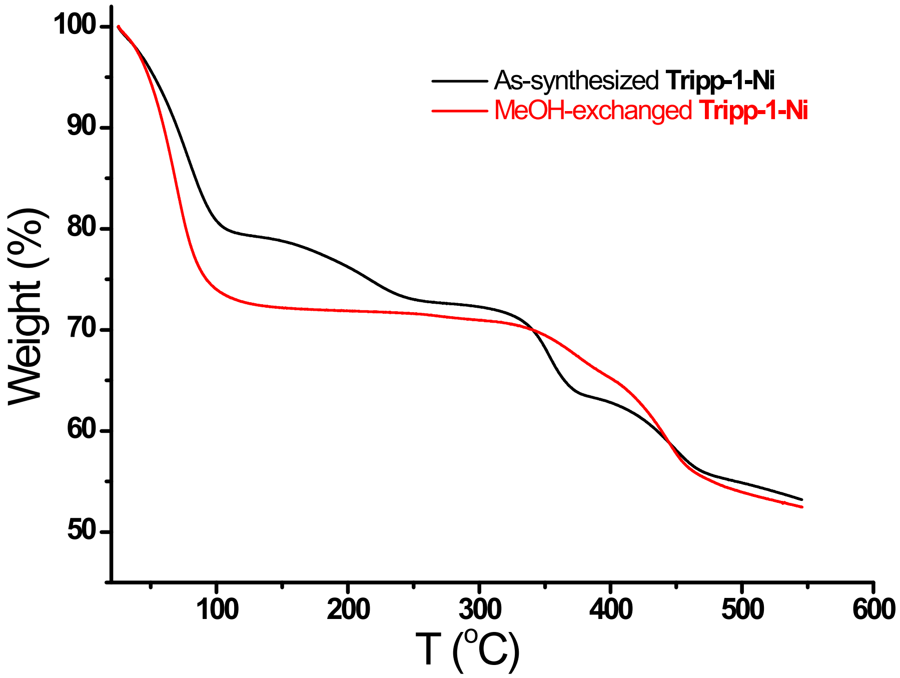

Figure S8. Thermo gravimetric analysis of Tripp-1-Co (top) and Tripp-1-Ni (bottom).

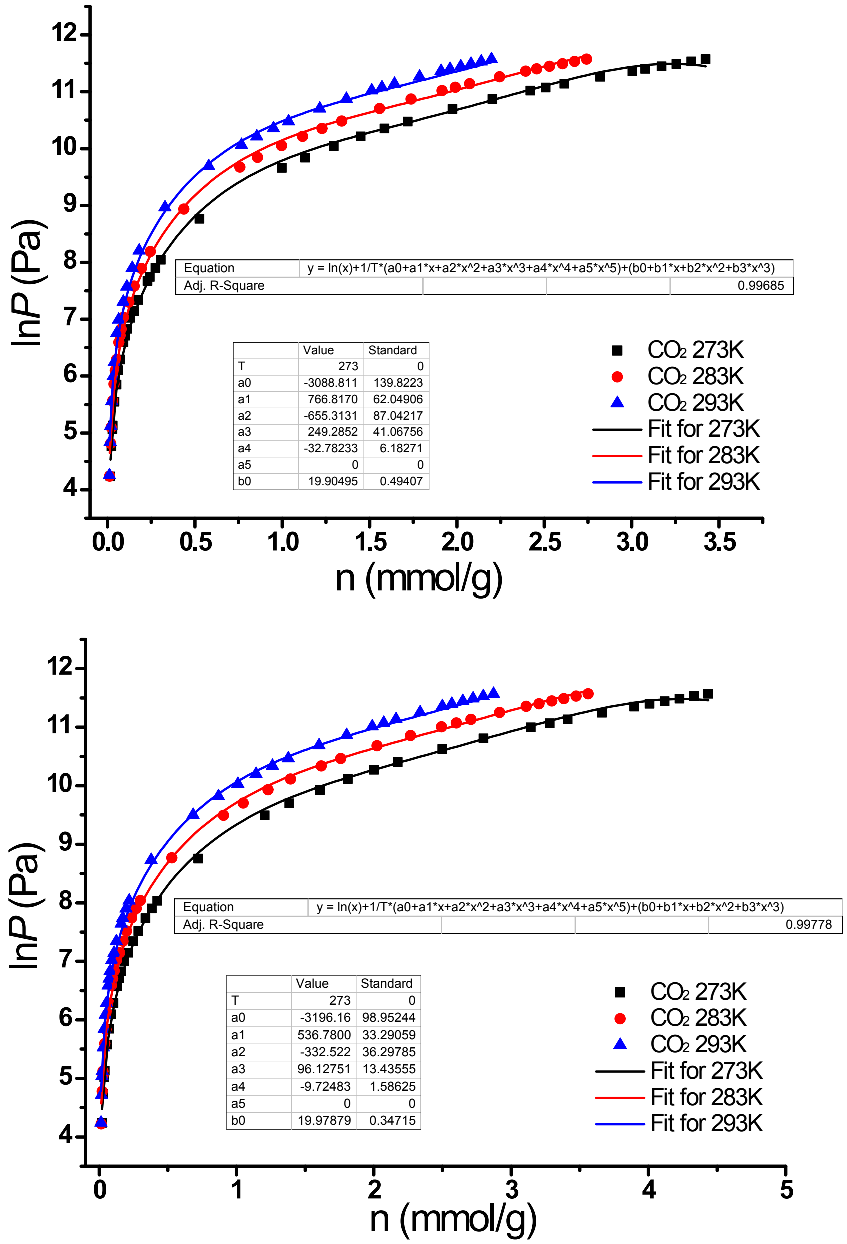

Figure S9. The Virial fit of CO_2 isotherms of Tripp-1-Co (top) and Tripp-1-Ni (bottom).

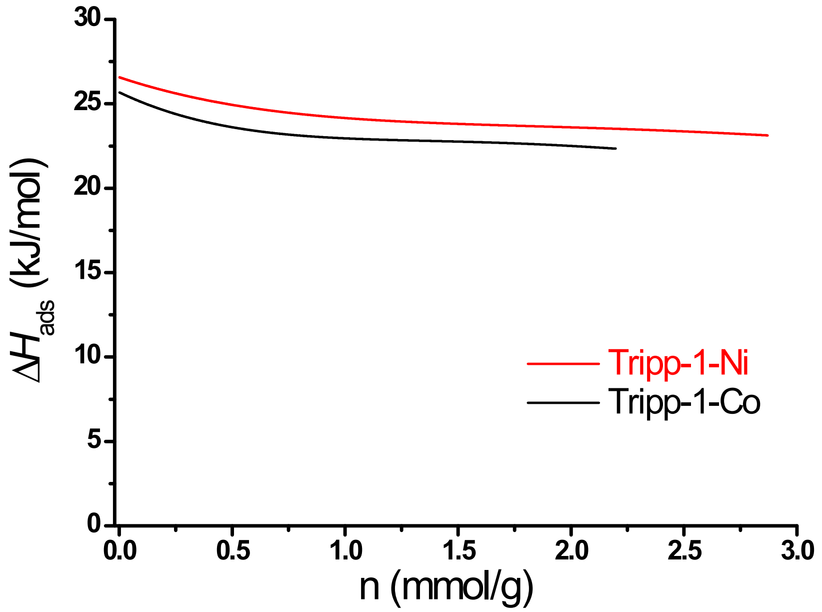

Figure S10. The isosteric heats of adsorption of Tripp-1-Co and Tripp-1-Ni.

Reference

1. Sheldrick, G. M. SHELXTL 6.12; Bruker Analytical Instrumentation: Madison, WI, 2000.
